# Characterization of DNA Methylation Associated Gene Regulatory Networks During Stomach Cancer Progression

**DOI:** 10.3389/fgene.2018.00711

**Published:** 2019-02-04

**Authors:** Jun Wu, Yunzhao Gu, Yawen Xiao, Chao Xia, Hua Li, Yani Kang, Jielin Sun, Zhifeng Shao, Zongli Lin, Xiaodong Zhao

**Affiliations:** ^1^School of Life Sciences, East China Normal University, Shanghai, China; ^2^Bio-ID Center, School of Biomedical Engineering, Shanghai Jiao Tong University, Shanghai, China; ^3^Department of Automation, Shanghai Jiao Tong University, Shanghai, China; ^4^Shanghai Center for Systems Biomedicine, Shanghai Jiao Tong University, Shanghai, China; ^5^Charles L. Brown Department of Electrical and Computer Engineering, University of Virginia, Charlottesville, VA, United States

**Keywords:** DNA methylation, gene regulation network, stomach cancer, tumor stages, system level

## Abstract

DNA methylation plays a critical role in tumorigenesis through regulating oncogene activation and tumor suppressor gene silencing. Although extensively analyzed, the implication of DNA methylation in gene regulatory network is less characterized. To address this issue, in this study we performed an integrative analysis on the alteration of DNA methylation patterns and the dynamics of gene regulatory network topology across distinct stages of stomach cancer. We found the global DNA methylation patterns in different stages are generally conserved, whereas some significantly differentially methylated genes were exclusively observed in the early stage of stomach cancer. Integrative analysis of DNA methylation and network topology alteration yielded several genes which have been reported to be involved in the progression of stomach cancer, such as *IGF2*, *ERBB2*, *GSTP1*, *MYH11*, *TMEM59*, and *SST*. Finally, we demonstrated that inhibition of *SST* promotes cell proliferation, suggesting that DNA methylation-associated *SST* suppression possibly contributes to the gastric cancer progression. Taken together, our study suggests the DNA methylation-associated regulatory network analysis could be used for identifying cancer-related genes. This strategy can facilitate the understanding of gene regulatory network in cancer biology and provide a new insight into the study of DNA methylation at system level.

## Introduction

DNA methylation plays a critical role in tumorigenesis through regulating oncogene activation and tumor suppressor gene silencing (He et al., 2008), and has raised extensive attention in the past decade. It has been shown that tumor initiation and development are associated with aberrant DNA methylation patterns, as documented in stomach cancer development (Tahara and Arisawa, 2015; Yamamoto et al., 2016). Aberrant DNA methylation pattern is the hallmark in the cancer genome (Baylin et al., 2000; Bergman and Cedar, 2013) and is involved in malignant progression (Jones et al., 2013). Although critically involved in malignancy, the implication of DNA methylation in tumorigenesis at system level is less characterized.

The gene regulatory network based analysis is regarded as a powerful way to understand the mechanism of tumorigenesis at system level (Kreeger and Lauffenburger, 2010), and various robust machine learning methods based gene regulatory network inference algorithms were proposed for such analysis (Haury et al., 2012; Slawek and Arodz, 2013; Wu et al., 2016). On the other hand, the rapid development of deep sequencing technologies promotes the generation of a tremendous amount of sequencing data, and an increasing number of network-based methods have been recently applied to understand the molecular mechanism of tumor formation and progression (Anglani et al., 2014; Yang et al., 2014; Bicker et al., 2015).

To further investigate the role of DNA methylation in tumorigenesis at system level, in this study we analyzed the DNA methylation-associated the topology dynamics of gene regulatory network in stomach cancer. We observed that although the DNA methylation patterns are generally conserved, the locus-specific DNA methylation patterns can be identified, especially in the early stage. Comparison of the topology of gene regulatory networks derived from different stages yielded several genes, such as *IGF2*, *ERBB2*, *GSTP1*, *MYH11*, *TMEM59*, and *SST*, of which the regulatory relationship is found to be most severely disrupted. To evaluate the biological relevance, we performed siRNA assay against *SST* in gastric epithelial cell line GES-1 and found that down-regulation of *SST* significantly promotes gastric cell proliferation. Collectively, these results suggest that the integrative analysis of DNA methylation and gene regulatory network across different stages of stomach cancer would be used to identify genes involved in stomach cancer initiation and development, and provides a new insight into the understanding of DNA methylation in carcinogenesis at system level.

## Results

### Probe-Gene Pairs Assignment

The DNA methylation datasets downloaded from the Cancer Genome Altas (TCGA) data portal were generated using two Illumina Infinium DNA methylation bead arrays (HM27 and HM450). Considering the incompleteness of DNA methylation data, we focused our study on the probes located in the gene promoter regions. Technically, more than one probes were generally designed for a given gene promoter region and it remains unclear which probe-hit methylated region actually affect the expression of the target gene. To address this issue, the distance and correlation criteria were used to assign the proper probes to a gene (See Materials and Methods for further details).

**FIGURE 1:**
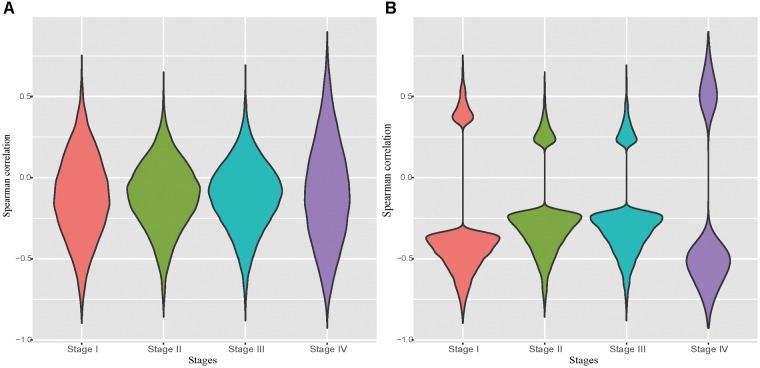
Distribution of correlations between the probe methylation level and the expression of target genes. **(A)**: Distribution of spearman correlation of all potential probe-gene pairs in the four stomach cancer stages. **(B)**: Distribution of spearman correlation of all significantly correlated potential probe-gene pairs in the four stomach cancer stages.

It has been well recognized that DNA hyper-methylation at the promoter region is associated with gene suppression (Bell et al., 2011; Jones, 2012). Due to the unavailability of DNA methylation data and the matched RNA-seq data in normal tissues, we examined the correlation between the pair of the expression level and the DNA methylation level of probes located in the promoter region of a given gene in each tumor stage. Not surprisingly, we observed that negatively correlated pairs outnumber the positive correlated ones (Figure 1A). Particularly, in the significantly correlated pairs we found that almost all probe-gene pairs were negatively correlated (Figure 1B). The probe-gene pair was assigned if the DNA methylation level of the probe and expression level of a gene are significantly negatively correlated in one of the four tumor stages. With these criteria, 10,777 probe-gene pairs, which consist of 9,830 probes and 7,546 genes, were defined and then used for the downstream analysis.

### Global Conserved and Locus Specific DNA Methylation Patterns Across Different Stomach Cancer Stages

**FIGURE 2:**
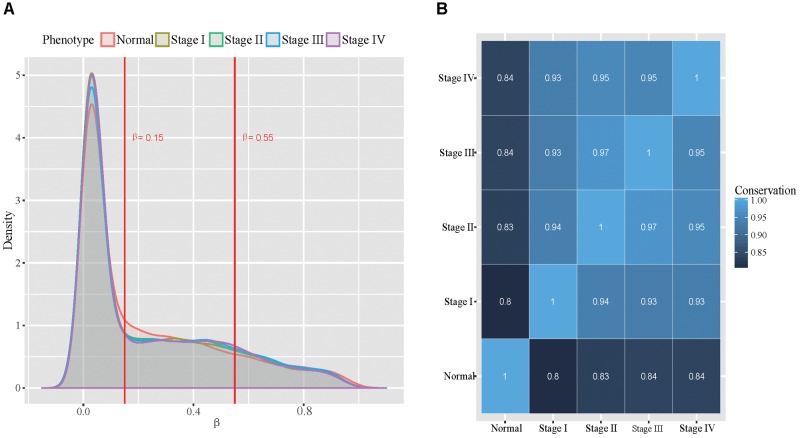
Global view of methylation patterns in all the five types. **(A)**: The distribution of methylation level across all the five phenotypes, where the two red lines represent the thresholds used for dividing the probes into three groups. **(B)**: The conservation between every two phenotypes.

With the selected probe-gene pairs, we firstly examined the global methylation patterns across all stomach cancer stages and the normal samples. We classified the probes into unmethylated, hemi-methylated and fully methylated groups using the approach similar to Lokk et al. (2012). To determine proper thresholds, we examined the distributions of the methylation level in all five phenotypes (Figure 2A). We found that the distributions of the methylation level in all five phenotypes are very similar. More than half of the probes were unmethylated and only about 15% probes were fully methylated in all samples. The dynamics in the methylation patterns across the five phenotypes was also analyzed. We found that the conservation between every two phenotypes was higher than 80% (Figure 2B), indicating that the DNA methylation patterns are globally conserved across all the five phenotypes. Additionally, we found that DNA methylation patterns are relatively more conserved in tumor stages.

**FIGURE 3:**
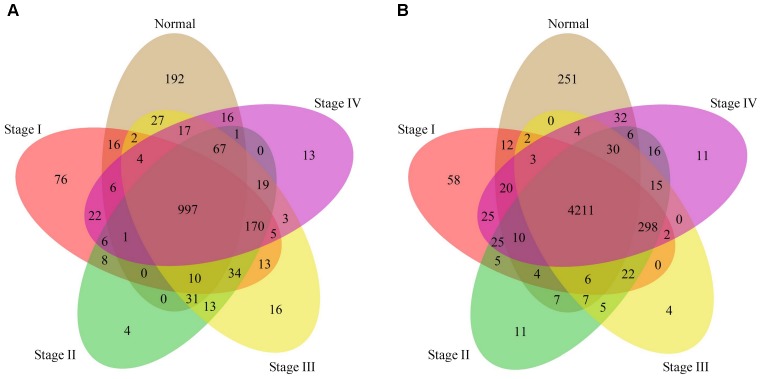
Venn diagrams of genes linked to the fully and unmethylated probes. **(A)**: The Venn diagram of fully methylated probe linked genes with respect to the five phenotypes. **(B)**: The Venn diagram of unmethylated probe linked genes with respect to the five phenotypes.

Although the overall patterns are considerably conserved, the phenotype-specific methylation presumably plays an important role in initiation and progress of stomach cancer. To test this presumption, we examined the presence of both the unmethylated and fully methylated probe-linked genes in the five phenotypes. Interestingly, we found that both the unmethylated and fully methylated probe-linked genes in normal samples were significantly more than those in tumor samples (Figure 3). We next performed gene ontology (GO) analysis of these genes with DAVID (Huang et al., 2009a,b). The results showed that the fully methylated probe-linked genes in normal samples were enriched in the GO items of defense response to bacterium and innate immune response (Supplementary Table S1), including *LPO* and *S100A8* which have been reported to be activated in the *H. pylori*-infected gastric mucosa (Semper et al., 2014; Zhuang et al., 2015).

**FIGURE 4:**
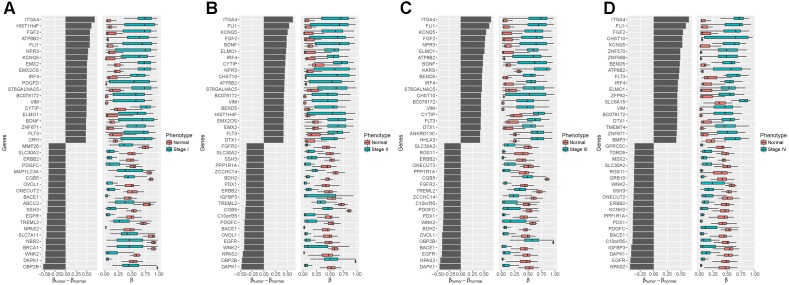
Differentialmethylation analysis between four tumor stages and the normal phenotype **(A)**: Stage I vs. Normal; **(B)**: Stage II vs. Normal; **(C)**: Stage III vs. Normal; **(D)**: Stage IV vs. Normal. Left: Mean difference between the methylation level in the tumor samples and the normal samples. Right: Distributions of methylation level, with black vertical lines showing medians. Top 20 of the largest positive and negative mean differences with an adjusted *p*-value less than 0.05 are shown.

To further understand the biological relevance of the DNA methylation in different stages of stomach cancer, we compared the samples in stages I–IV with the normal samples and identified the significantly differentially methylated probes. We found 1,059, 716, 673 and 635 genes linked to significantly differentially methylated genes in stages I–IV samples, respectively. The top 20 significantly differentially methylated probe linked genes with largest positive and negative mean differences were shown in Figure 4, in which we found that several oncogenes and tumor suppressor genes were at the top of the lists (positive and negative directions, respectively) in all four tumor stages, including *ITGA4*, *FGF2*, *FLI1*, *EGFR*, *ERBB2*, VIM, and *DAPK1*. *ITGA4* encodes a member of the integrin alpha chain family that may play a role in cell motility and migration, and the promoter of *ITGA4* was reported to be hyper-methylated in various cancers, such as colorectal cancer (Gerecke et al., 2015), breast cancer (Lian et al., 2012) and gastric cancer (Kim et al., 2009). *DAPK1*, a positive mediator of gamma-interferon induced programmed cell death, was reported to be fully hypo-methylated or up-regulated in several types of cancer, including fistula associated mucinous type anal adenocarcinoma (Sen et al., 2010), nasopharyngeal carcinoma (Luo et al., 2011) and gastric cancer (Zhang et al., 2006).

**FIGURE 5:**
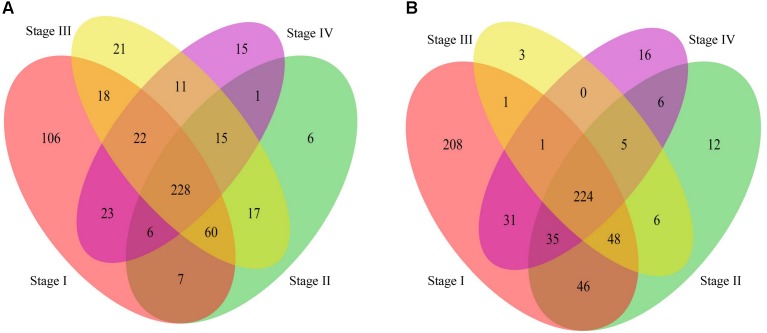
Venn diagram of genes linked to the differentially methylated probes in stage I to IV compared to the normal phenotype. **(A)**: The Venn diagram of genes linked to the hyper-methylated probes. **(B)**: The Venn diagram of genes linked to the hypo-methylated probes.

The Venn diagram of genes with significantly differentially methylation was shown in Figure 5. We found that most genes were shared by stages II – IV except in stage I. The GO analysis (Supplementary Table S2) shows that the commonly hyper-methylated probe linked genes are mainly involved in carcinogenesis related biological processes, such as cell motion, cell death and cell migration. While the commonly hypo-methylated probe linked genes are mainly involved in development and differentiation biological processes (Supplementary Table S3). We also found some genes exclusively present in stage I, suggesting that they are presumably associated with the early stage of stomach cancer. The GO analysis results revealed that both the specifically hyper-methylated genes and the specifically hypo-methylated genes are involved in cell adhesion and transmembrane transport. The difference is that the genes linked to the specifically hyper-methylated probes are particularly involved in eating behavior and positive regulation of appetite (Supplementary Table S4), while the genes linked to the specifically hypo-methylated probes are particularly involved in immune response, response to bacterium and negative regulation of Wnt signaling pathway (Supplementary Table S5).

### Regulation Gain or Loss Induced by DNA Methylation Alteration

DNA methylation is one of the key epigenetic mechanisms involved in regulation of gene expression. To further understand the role of DNA methylation alteration during the stomach cancer development, we constructed a DNA methylation associated gene regulatory network for each phenotype and analyzed the topology differences among these networks.

**FIGURE 6:**
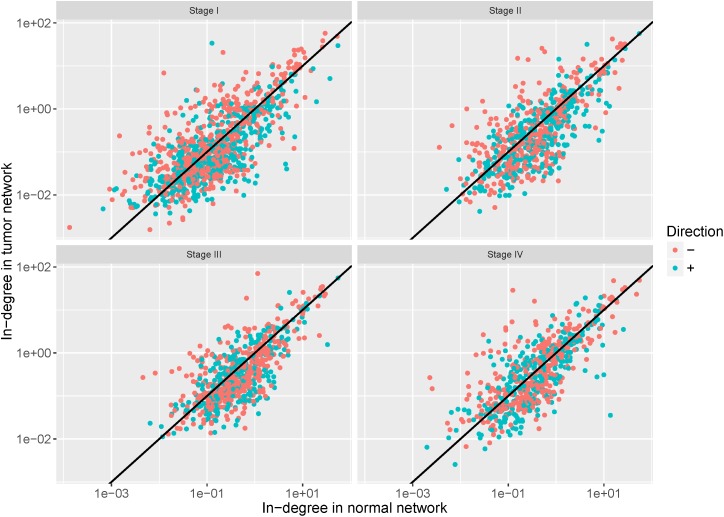
In-degree of each target gene in each network pair. The red dots represent the retained genes that satisfy the assumption that hyper-methylation may cause loss of regulation and hypo-methylation may cause its gain. The blue dots represent genes discarded in the further analysis.

To examine the regulation alteration affected by the DNA methylation changes, we screened the target genes based on the assumption that the hyper-methylation leads to the reduction of affinity between the TFs and the binding regions and then may cause the loss of regulation while the hypo-methylation causes its gain (Yao et al., 2016). We calculated in-degree for each target gene and the genes with in-degree increase linked to hypo-methylated probes (in-degree decrease genes linked to hyper-methylated probes) were retained. The in-degree of each target gene in each network pair were shown in Figure 6. After filtering, 57%, 52%, 59%, and 54% of target genes were retained in stages I–IV, respectively.

To further investigate the regulation alteration in four tumor stages compared to the normal phenotype, we constructed the differential regulatory networks by subtracting the normal weight matrix from the tumor weight matrixes. The regulation relationship with the absolute weight difference ranking top 1,000 was regarded as true alterations. Finally, for each tumor stage we obtained a differential regulatory network consisting of 1,000 edges that point to 172, 172, 189, and 176 target genes in the four tumor stages. The numbers of edges pertaining to gain or loss of regulation were listed in Table 1, in which we observed that the gain number is larger than the loss number in each of the four tumor networks.

**Table 1:** Numbers of gain and loss of regulation in each of the four tumor related networks.

	Stage I	Stage II	Stage III	Stage IV
Loss	308	408	464	419
Gain	692	592	536	581

**FIGURE 7:**
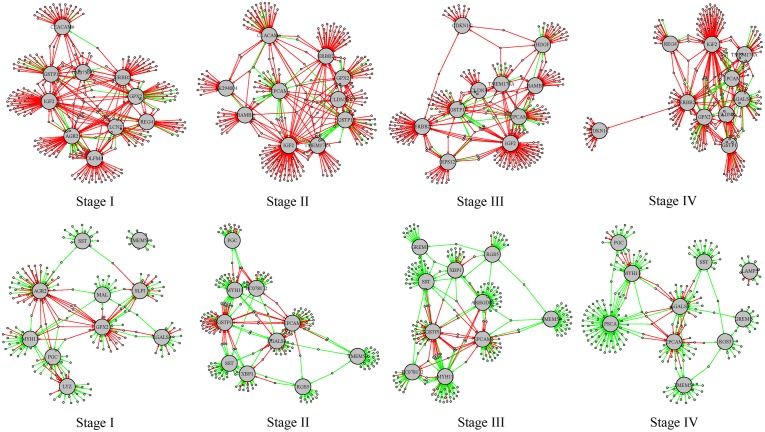
Subgraphs involving the top 10 target genes with the largest number of regulations gained or lost stages I–IV. The red edges represent the regulations gained in the tumor phenotype and the green edges represent regulations lost in the tumor phenotype. The larger gray nodes are target genes and the smaller gray dots are transcription factors involved. The top 4 subgraphs are regulation relationships involving the top 10 target genes with the largest number of regulations gained; the bottom 4 subgraphs are regulation relationships involving the top 10 target genes with the largest number of regulations lost.

For the differential regulatory network in stages I–IV, we ranked the target genes according to the number of gained or lost regulation, respectively. We found several genes were at the top in all the tumor stages. The top 10 target genes (listed in Supplementary Table S6) with the largest number of regulation alteration were shown in Figure 7. In these subgraphs we found that *IGF2*, *ERBB2*, and *GSTP1* rank top in the largest number of regulation gained in all the four differential regulatory networks, and *MYH11*, *SST*, and *TMEM59* rank top in the largest number of regulation lost in all the four differential regulatory networks. *IGF2* is an imprinting gene and plays an essential role in the embryonic development. However, activation of *IGF2* stimulates the proliferation of tumor cells and prevents damaged cells from being destroyed. It was reported that overexpression of *IGF2* plays an important role in carcinogenesis of diffuse type gastric cancer (Wu et al., 1997). *MYH11* belongs to a group of proteins called myosins, which are involved in cell movement and the transport of material within and between cells. It was reported that *MYH11* is not expressed in gastric cancer cell lines (Saeki et al., 2015) and down-regulated *MYH11* correlates with poor prognosis in stage II and stage III colorectal cancer (Wang et al., 2014). These results indicate that the methylation-mediated network analysis facilitates the identification of the key genes involved in tumorigenesis.

**FIGURE 8:**
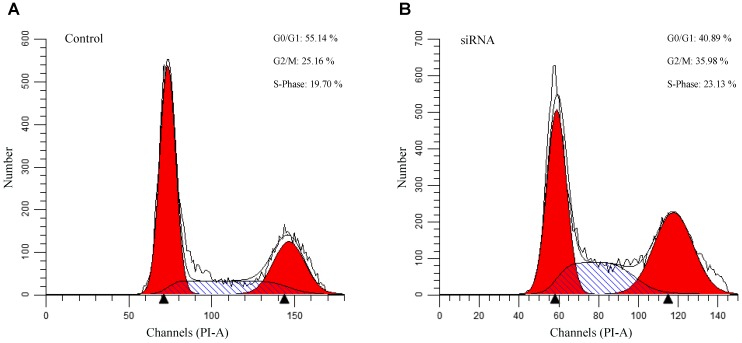
Flowcytometry analysis of the *SST* knockdown gastric cells. **(A)**: Cell cycle analysis of control siRNA GES-1. **(B)**: Cell cycle analysis of *SST* knockdown siRNA GES-1.

To evaluate the authenticity of the genes identified through our network analysis, we performed a siRNA assay against *SST* in gastric epithelial cell line GES-1. Comparing with the control, we found that *SST* suppression results in an increase of cells in S and G2/M phases and the decrease of cells in the G0/G1 phase (Figure 8), indicating that *SST* down-regulation promotes cell proliferation. From the results, we found that inhibition of *SST* promotes cell proliferation, which suggests that DNA methylation-associated *SST* suppression possibly contributes to the gastric cancer progression.

## Discussion

It has been recognized that aberrant DNA methylation play an import role in tumorigenesis. However, the implication of DNA methylation in gene regulatory network is less characterized. Thus, we performed an integrative analysis of DNA methylation and gene regulatory network with the RNA-seq and DNA methylation data to understand the role of DNA methylation change in the gene regulatory network alteration across different stomach cancer stages.

We first assigned a gene with appropriate probes according to both the location information and correlation relationship. We found that the DNA methylation pattern was global conserved across all phenotypes except some locus specific DNA methylation patterns in the normal phenotype. The differential methylation analysis was also performed to identify the significantly differentially methylated genes in each tumor stage samples. Interestingly, we found more specific alterations in the stage I phenotype compared to the other tumor stages and the GO analysis results showed that these genes are particularly involved in the biological processes closely related to the cancer initiation.

To identify the gene regulation alteration affected by the DNA methylation change, we constructed a DNA methylation associated gene regulatory network in each phenotype and subtracted the normal network from the four tumor networks, respectively. The differential network analysis results showed that the number of regulations gained was larger than that of regulations lost in each of the four tumor networks. We ranked the target genes according to the number of altered regulations and obtained several genes that rank top in all the tumor stages. For example, *IFG2*, *ERBB2*, and *GSTP1* ranked top in the largest number of regulation gain and *MYH11*, *TMEM59*, and *SST* ranked top with the largest number of regulations loss. To examine the biological relevance of the genes identified, we selected *SST* for functional evaluation. We found that inhibition of *SST* can significantly promote cell proliferation, which suggests that down-regulation of *SST* is involved in stomach cancer progression.

In brief, our study demonstrated that integrative analysis of the regulatory network and DNA methylation allows identifying cancer-related gene. The strategy proposed here provides new insight into understanding of the role of DNA methylation in disease at system level.

## Materials and Methods

### Data Collection and Differentially Methylated Sites Identification

The DNA methylation data, gene expression data and clinical data were downloaded from TCGA data portal. The DNA methylation data consist of 302 samples, which were generated using two Illumina Infinium DNA methylation bead arrays, HumanMethylation27 (HM7) and HumanMethylation450 (HM450). The HM27 array contains 27,578 probes that target CpG sites located in proximity to the transcription start sites and the HM450 array contains 482,421 probes that target CpG sites throughout the genome. For ease of description, in the following sections of this article we used probes to represent the corresponding CpG sites.

As neither the HM27 nor the HM45 data contains enough samples for analysis for each phenotype, we only took probes located in gene promoters into account even though the DNA methylation of transcriptional enhancers was also reported to be closely associated with carcinogenesis (Aran and Hellman, 2013). We adopted the strategy mentioned in a previous report (Bass et al., 2014) to preprocess the DNA methylation. Briefly, the probes shared by both the HM27 and HM450 platforms were selected, and the probes that overlap with SNPs, repeat and have any “NA”-masked data points were removed. The probes that hit X and Y chromosomes were also removed. After that we obtained 19,736 probes for further analysis. The gene expression data of 272 samples and 26,540 genes were generated using RNA-seq. The DNA methylation samples and the gene expression samples were further divided into five phenotypes, which are normal and tumor stages I–IV, according to the clinical data. Sample numbers for all phenotype are listed in Table 2.

**Table 2:** Number of samples in each phenotype for the RNA-seq and DNA methylation data.

	Normal	Stage I	Stage II	Stage III	Stage IV
RNA-seq	29	35	93	92	23
DNA methylation	27	37	102	111	25
Matched	0	35	93	92	23

As we did not expect all cases to be from a single molecular subtype, and we sought to identify methylation changes within cases from the same molecular subtype. To identify the significantly differentially methylated probes, we excluded the 10% of samples with the lowest methylation and 10% samples with the highest methylation for each probe and the Wilcoxon Rank Sum test was used to measure the significance. Probes with a BH-adjusted *p*-value less than 0.05 and an absolute methylation difference greater than 0.2 were regarded as significantly differentially methylated.

### Assigning DNA Methylation Sites to the Target Gene

In general more than one DNA methylation probes of the DNA array were designed for a given gene promoter region. Thus, it remains unclear which probes actually affect the expression of the target gene. To address this issue, we used two criteria to assign the DNA methylation probes for each gene. We initially assigned a probe to a gene if the probe located in the promoter region of the gene. The promoter region of a gene is defined as ±2 kb region around the transcription start site of the gene. The relationship between a probe and a gene is then confirmed with the aid of gene expression based on the evidence that DNA methylation can repress the transcription when it occurs in the promoter region. The samples with matched gene expression data and methylation data were used for the analysis. For each candidate, we tested the significance of the correlation between the DNA methylation level of the probe and expression level of the gene. The Spearman’s coefficient was used as the measure of correlation. The correlation significance was obtained with *t*-test and the t statistic was calculated as:

$$t=\frac{r\sqrt{n-2}}{1-r^{2}},$$

where *r* is the correlation between the methylation and gene expression and *n* is the number of samples. The probe-gene pairs were finally confirmed if the BH-adjusted *p*-value is less than 0.05 and the correlation less than zero.

### DNA Methylation Associated Gene Regulatory Network Construction

To construct the DNA methylation associated gene regulatory network, the potential TFs which maybe bind to the DNA methylated regions should be identified. We first obtained JASPAR-2014 motif position weight matrices (PWMs) and ENCODE motif PWMs from the R package motifDb and 2,182 motif PWMs were used for further analysis (ENCODE Project Consortium, 2004; Mathelier et al., 2014). The potential TFs bound to each target gene were predicted according to sequence affinity. We used FIMO (Grant et al., 2011) to scan a ±100 bps sequence around each probe in search for instances of the selected PWMs. A TF was regarded a potential regulator of a probe-linked genes if the *p*-value of its motif is less than 1*E*-4. However, a high sequence affinity just indicated that the TF has a high opportunity to bind to the regulatory region. It was unclear whether the gene relate to the regulatory element is actually bound by the TF.

To measure the confidence of such regulation relationship, we assigned a weight to the edge outgoing from a potential TF to the target gene using our previously proposed gene regulatory network inference method (Wu et al., 2016) with the RNA-seq data. Briefly, we assumed that the expression level of target gene can be formulated by an unknown function of the expression of TFs. We first solved the individual regression problem with the guided regularized random forest algorithm, and then a q-norm normalization was employed to reduce the bias among different regression results and the final results were obtained through refining the previous results according to the sparsity property of large scale gene regulatory networks.

### RNA Interference and Cell Cycle Analysis

RNA interference assays were performed as reported previously. SiRNAs for *SST*, or negative control, were synthesized by Shanghai GenePharma Co., Ltd. Cells were transfected with *SST* siRNA or control siRNA using Lipofectamint^TM^ 2000 Transfection Reagent (11668027, Invitrogen) according to the manufacturer’s protocol. To measure the efficacy of the gene knockdown, the quantitative real-time reverse transcription polymerase chain reaction (RT-qPCR) was used. Total RNA was extracted using TRIzol Reagent (15596-018, Invitrogen) and resuspended in RNase free water. Reverse transcription of 1 μg RNA was performed using the oligo-dT primer and SuperScrip^®^III Reverse Transcriptase (18080-044, Invitrogen) according to the manufacturer’s protocol. Expression levels were determined by real-time PCR using ABI step one plus (Applied Biosystems, United States). **β**-actin was used as a control gene for normalization. The relative level of mRNA was calculated as 2^−ΔΔCt^ (means ± SEM, *n* = 3). The *SST*-targeting siRNA, primer sequences and the RT-qPCR results were provided in Supplementary Table S7.

## Author Contributions

XZ and JW conceived and designed the project. JW wrote the manuscript. YG and YK performed the experiments. JW, YX, CX, and HL performed the analysis and interpretation of data. JS, XZ, ZL, and ZS made a substantial contributions to the design and revisions of the manuscript. All authors have read and approved the final version of the manuscript.

## Conflict of Interest Statement

The authors declare that the research was conducted in the absence of any commercial or financial relationships that could be construed as a potential conflict of interest.
