# Supplementary material for: Characterization of DNA Methylation Associated Gene Regulatory Networks During Stomach Cancer Progression
Source: Front Genet. 2019 Feb 4;9:711. doi: 10.3389/fgene.2018.00711 (PMC6369581; doi:10.3389/fgene.2018.00711)
Supplement: Supplementary file 7 [file Table_7.DOCX]

A: The sequences of *SST*-targeting siRNA and negative control.

| siRNA |  |
| --- | --- |
| STT | GUCCAUAUCAGACCUCUGATT |
|  | UCAGAGGUCUGAUAUGGACTT |
| Negative control | UUCUCCGAACGUGUCACGUTT |
|  | ACGUGACACGUUCGGAGAATT |

B: The primer sequences for RT-qPCR

| qPCR primer |  |
| --- | --- |
| beta-actin F | GCAAGCAGGAGTATGACG |
| beta-actin R | CAAGAAAGGGTGTAACGC |
| STT-F | TCAAACCCGGCTATGGCAC |
| STT-R | TTAGGGAAGAGAGATGGGGTGT |

C: SST expression is decreased in the mRNA levels after treatment with *SST*-targeting siRNA (siSST) or control siRNA (siControl).
